# The characteristics of the tumor immune microenvironment in colorectal cancer with different MSI status and current therapeutic strategies

**DOI:** 10.3389/fimmu.2024.1440830

**Published:** 2025-01-14

**Authors:** Qingzhe Wang, Min Yu, Shuang Zhang

**Affiliations:** ^1^ Department of Targeting Therapy and Immunology, Cancer Center, West China Hospital, Sichuan University, Chengdu, Sichuan, China; ^2^ Division of Thoracic Tumor Multimodality Treatment, Cancer Center, West China Hospital, Sichuan University, Chengdu, Sichuan, China

**Keywords:** colorectal cancer, tumor microenvironment, immune checkpoint inhibitors, deficient mismatch repair, proficient mismatch repair, microsatellite instable, microsatellite stable

## Abstract

Colorectal cancer (CRC) remains a significant cause of cancer-related mortality worldwide. Despite advancements in surgery, chemotherapy, and radiotherapy, the effectiveness of these conventional treatments is limited, particularly in advanced cases. Therefore, transition to novel treatment is urgently needed. Immunotherapy, especially immune checkpoint inhibitors (ICIs), has shown promise in improving outcomes for CRC patients. Notably, patients with deficient mismatch repair (dMMR) or microsatellite instability-high (MSI-H) tumors often benefit from ICIs, while the majority of CRC cases, which exhibit proficient mismatch repair (pMMR) or microsatellite-stable (MSS) status, generally show resistance to this approach. It is assumed that the MSI phenotype cause some changes in the tumor microenvironment (TME), thus triggering antitumor immunity and leading to response to immunotherapy. Understanding these differences in the TME relative to MSI status is essential for developing more effective therapeutic strategies. This review provides an overview of the TME components in CRC and explores current approaches aimed at enhancing ICI efficacy in MSS CRC.

## 1 Introduction

Colorectal cancer (CRC) is the third most commonly diagnosed cancer, accounting for 10.2% of all cases globally, and it is the second leading cause of cancer mortality, responsible for 9.2% of cancer death (1). Standard treatments for CRC include surgery, chemotherapy, radiotherapy, and targeted therapy, such as epidermal growth factor receptor (EGFR) inhibitors (e.g., cetuximab) and vascular endothelial growth factor receptor (VEGFR) inhibitors (e.g., bevacizumab) (2, 3). Unfortunately, the prognosis for patients with metastatic CRC remains poor, with over 80% of these patients succumbing to the disease within 5 years of diagnosis (4, 5).

Immunotherapy, which harnesses and modulates the patient’s immune system to combat cancer, is a promising treatment avenue. Immune system distinguishes self from non-self through the interaction between T-cell receptors (TCRs) and peptides by major histocompatibility complex (MHC) molecules on the surface of all cells, including tumor cells (6, 7). Co-stimulatory or co-inhibitory ligands can modulate the TCR-MHC signaling pathway, a mechanism often exploited by tumor cells to evade immune surveillance (8, 9). Immune checkpoint inhibitors (ICIs), which target co-inhibitory pathways such as cytotoxic T lymphocyte antigen 4 (CTLA4) and programmed cell death 1 (PD-1) on T cells, as well as programmed death ligand 1 (PD-L1) on tumor and immune cells, can restore T-cell function and enhance antitumor immunity (10). While ICIs have shown impressive efficacy in cancers like lung cancer and melanoma, their success in CRC varies significantly with disease stage and molecular subtype, underscoring the molecular heterogeneity of CRC.

Microsatellites, defined as short series of DNA repeats, are susceptible to occur replication errors typically corrected by the mismatch repair (MMR) system. Patients with the dMMR phenotype often exhibit MSI in tumor microenvironment (TME), a feature present in approximately 15% in CRC cases (11). Dysfunctional MMR systems lead to higher tumor mutational burdens (TMBs) in MSI tumors compared to microsatellite stable (MSS) tumors, generating a more robust antitumor immune response (12). Previous studies have shown that patients with dMMR/MSI-high (MSI-H) CRCs exhibit an objective response rate (ORR) of approximately 60% when treated with ICIs (13, 14), leading the FDA to approve ICIs for dMMR/MSI-H CRC patients (10). In contrast, MSS CRCs—which represent approximately 85% of metastatic CRC cases—demonstrate an ORR of only 5%–10% (10).

Immunophenotyping and antigenome meta-analyses reveal that MSI CRCs have a TME enriched in CD4^+^ and CD8^+^ T cells and depleted of myeloid-derived suppressor cells (MDSCs), underscoring the critical role of the TME in determining immunotherapy efficacy (15). However, among MSI tumors, those with mutations in genes involved in antigen processing and presentation, such as B2M, TAP1, TAP2, NLRC5, and RFX5, may still evade immune surveillance and show limited response to immunotherapy, which provides a clue of immunotherapy resistance of MSS phenotype (16–18). The mechanisms underlying immunotherapy resistance in these cases remain unclear and warrant further investigation.

## 2 Tumor immune microenvironment in CRC

The TME, where tumors reside, is composed of immune cells, blood vessels, extracellular matrix (ECM), fibroblasts, and various signaling molecules (19, 20). Interactions between malignant and nonmalignant cells within TME are crucial for cancer development, progression, metastasis, and therapeutic outcome (21–23). Notably, the composition and spatial distribution of TME differ between MSS and MSI CRCs (10). **Table 1** provides a summary of the characteristics of cellular components within the TME.

**Table 1:** Characteristics of cellular components in TME.

Immune cells	Inducible factors	Productions	Expression preference	Prognosis
CD8^+^ TIL	IL-2, IL-4, IL-7, IL15, IFN-γ	Perforin, granzymes, granulysin, Fas ligand, TNF-α	MSI	Good
Th1	IL-12 and IFN-γ	IFN-γ, TNF-α,	MSI	Good
Th17	TGF-β and IL-6	17A, IL-17F, IL-21, IL-22, TGF-β, IFN-γ, TNF-α	MSS	Poor
Treg	TGF-β and IL-12	TGF-β, IFN-γ, IL-10	MSS	Polarize
γδ T cell	IL-8 and VEGF	IFN-γ (Vδ1 T), IL-17 (Vδ2T)	MSI	Polarize
MDSC	PGE2, IL-6, VEGF, IL-1β, IL-17, TGF-β	IL-10, TGF-β, iNOS, ARG1	MSS	Poor
TAM	M1: IFN-γ, LPS, TNF-α	M1: IL-6, IL-23, ROS	M2:pMMR	M1: good;
	M2: IL-4, IL-10, IL-13, glucocorticoid	M2: IL-10, IL-1β		M2: poor
TAN	N1: IFN-γ	N1: IL-12, IL-18, IL-17, IL-23 and TNF-α	MSS	Polarize
	N2:IL-8, TGF-β	N2: arginase, MMP-9, VEGF		
NK cells	IL-12, IL-15, and IL-18	IFN-γ, IL-10, IL-13, TNF-β, and GM-CSF	MSI	Good
DCs	Flt3L	IL-12, IL-6, TNF-α, and IL-1b	MSI	Good

### 2.1 T cells

T cells, the most abundant immune cells in TME, are traditionally categorized into two main subsets: CD8^+^ and CD4^+^ T cells, each equipped with TCRs composed of an alpha and a beta chain (24). Tumor antigens are initially recognized and processed by antigen-presenting cells (APCs), such as dendritic cells, and subsequently presented on MHC class I and MHC class II molecules to CD8^+^ T cells and CD4^+^ T cells, respectively, collectively driving a robust antitumor immune response.

#### 2.1.1 CD8^+^ T cell

CD8^+^ cytotoxic T lymphocytes (CTLs) are the primary T-cell subset responsible for directly killing tumor cells in the antitumor immune response. These cells are activated by cytokines such as interleukin-2 (IL-2), IL-4, IL-7, IL-15, and interferon-γ (IFN-γ). In TME, chemokines like CCL5 and CXCL9/10/11 bind to CXCR3 on CD8^+^ T cells, recruiting them to the TME, where they exert cytotoxic effects by secreting perforin, granzymes, granulysin, Fas ligand, and tumor necrosis factor α (TNF-α) (25–28). CD8^+^ T-cell infiltration is known to correlate strongly with improved patient survival and reduced recurrence risk, particularly in advanced cases (T4, N1-2), regardless of DNA MMR deficiency, POLE mutation, or chromosomal instability status (29, 30).

In MSS CRC, immune evasion often occurs through reduced CD8^+^ T-cell infiltration in the tumor center and functional exhaustion of these cells. In CRC, CD8^+^ T-cell density is generally lower in the tumor center than at the invasive margin, with higher central infiltration levels associated with better overall survival (31). Moreover, while CD8^+^ T-cell densities in stroma were similar in both MSS and MSI CRCs, as well as that in the tumor glands, MSI-H tumors tend to exhibit greater CD8^+^ T-cell density in both the tumor core and invasive margin (32). Studies have shown that CD8^+^ T cells in MSI-H tumors are more abundant and exhibit enhanced cytotoxic activity (33–37).

In MSS CRC, TIM-3^+^PD-1^+^CD8^+^CILs often accumulate with exhaustion phenotype, highlighting the role of co-inhibitory signal in T-cell dysfunction (38). Research has indicated that cholesterol accumulation in TME may exacerbate endoplasmic reticulum (ER) stress, which upregulates inhibitory receptors such as PD-1, 2B4, TIM-3, and LAG-3 on CD8^+^ T cells. Notably, inhibition of the ER stress sensor XBP1 or reduction of cholesterol has been shown to restore CD8^+^ T-cell antitumor activity (39). Furthermore, high level of IL-2 in TME can enhance inhibitory receptors’ expression and reduce cytokine and effector molecule production in CD8^+^ T cells through the activation of the STAT5 pathway (40). TOX, a transcription factor closely associated with T-cell exhaustion, is necessary for T-cell persistence within tumors; TOX deletion impairs T-cell survival, suggesting that T-cell exhaustion may serve as a regulatory mechanism to prevent overactivation and subsequent cell death in chronic immune responses (41).

#### 2.1.2 CD4^+^ T cell

CD4^+^ helper T lymphocytes are crucial players in the immune response, not only activating other immune cells like B cells and cytotoxic T cells but also differentiating into subsets with diverse functions, including T helper (TH) 1 cells, TH2 cells, TH17 cells, TH9 cells, follicular helper T (Tfh) cells, and regulatory T (Treg) cells (42). Each of these subsets has distinct surface markers, transcription factors, polarizing cytokines, functions, and cytokine profiles, as detailed in prior comprehensive review (24). Unlike their CD8^+^ counterparts, CD4^+^ memory T cells are persistently present, which is vital for a sustained response to tumor antigens (43). Within TME, each CD4^+^ subset plays unique and sometimes contrasting roles, interacting in complex ways. For example, TH1-derived IFN-γ and TH2-derived IL-4 mutually inhibit each other’s expression (44). TH2 and TH9 lineages, despite sharing transcriptional pathways like STAT6, exert opposing effects within the TME, with TH2 generally supporting tumor progression, whereas TH9 promotes antitumor immunity (44).

TH1 cells are associated with favorable outcomes in CRC, as they can enhance cancer cell apoptosis, inhibit angiogenesis, and recruit toxic CD8^+^ T cells (45, 46). Compared with the MSS phenotype, MSI CRCs have a higher density of TH1 cells, underscoring their antitumor role within TME (32, 47). TH1 cells have also been reported to reshape the tumor-associated myeloid cell network, promoting interferon-activated antigen presentation and iNOS-expressing tumoricidal effector phenotypes, which together indirectly eradicates interferon-unresponsive and MHC-deficient tumors (48). TH2 cells, traditionally involved in immunity to parasites and allergic diseases, have recently been observed to exhibit antitumor activity through cytokines such as IL-4 and IL-5 (49). However, the TH2 cluster expression does not correlate with CRC patient prognosis (45). The role of TH2 cells in the TME remains complex and warrants further investigation to better understand their potential contributions to CRC outcomes.

TH17 cells play a crucial role in mucosal defense by producing various pro-inflammatory cytokines, including IL-17A, IL-17F, IL-21, IL-22, and TNF-α (50). They are activated through TGF-β and IL-6 via the p-STAT3 and RAR-related orphan receptor-γt (ROR-γt)-dependent pathways, while IL-23 serves as a growth and stabilizing factor (51). Within TME, TH17 cells are recruited by chemokines CCL4, CCL17, CCL20, and CCL22 (52). Research has demonstrated the pro-tumorigenic role of TH17 cells through several mechanisms (1): reducing CD8^+^ T-cell infiltration by inhibiting the production of chemokines CXCL9 and CXCL10 and downregulating CXCR3 expression via the IL-17A/STAT3 signaling pathway; (2) creating an immunosuppressive TME by recruiting and accumulating MDSCs; (3) impeding antigen presentation by dendritic cells (DCs) to CD4^+^ T cells through upregulation of nitric oxide (NO) levels; (4) enhancing the immunosuppressive capacity of mesenchymal stem/stromal cells (MSCs) and macrophage, thus facilitating tumor progression; and (5) inducing the expression of immunosuppressive mediators, including IL-6, TGF-β, and CCR6 (53–58). Nearly two-thirds of primary sporadic CRCs exhibit increased levels of TH17 cells, which is associated with poor prognosis (45). Compared to MSI-H tumors, MSS tumors show a higher proportion (approximately 40%) of TH17-cell-infiltrated phenotype (47, 59). Among patients with MSS CRC, those with lower IL-17A^+^ cell infiltration have shown better tumor control rates following anti-PD-1 immunotherapy (60), making IL-17 a potential target. Interestingly, owing to the presence of different IL-17 subtypes, TH17 cells can exhibit dual roles. Besides a protumor role, IL-17F has been shown to have antitumoral effects, and intraepithelial IL-17^+^ cell infiltration is positively associated with an improved prognosis (61, 62). Additionally, TH17 lineage can exert antitumorigenic effects by recruiting immune cells such as DCs, CD4^+^ T cells, and CD8^+^ T cells (52). This dual functionality might explain the association between tumor-infiltrating TH17 cells and favorable prognosis in cancers like oral squamous cell carcinoma, gastric cancer, and cervical cancer (63–65).

#### 2.1.3 Regulatory T cells

Regulatory T cells (Tregs), characterized by high expression of CD25 and the transcription factor forkhead box protein P3 (FOXP3), play a crucial role in maintaining tolerance to self-antigens and immune homeostasis through production of TGF-β and IFN-γ, as well as direct cell-to-cell contact (66, 67). Within CRC TME, Tregs are induced by TGF-β and IL-12 and recruited by chemokines such as CCL1, CCL3, CCL4, CCL17, CCL20, and CCL22 (68, 69). The conversion of naive and memory conventional T cells to Tregs is facilitated by immature APCs and MDSCs (70). Tregs suppress antitumor immunity by inducing apoptosis in CD8^+^ T cells, producing IL-10 to inhibit NK cells and conversion of TH17 and TH1 cells, thus creating an immunosuppressive TME that supports CRC progression (71, 72). Elevated levels of Tregs have been detected in peripheral blood, tumor-draining lymph node, and tumor site of CRC patients (68, 71, 73). In particular, intratumor Tregs demonstrate higher suppressive activity (74). Compared to MSI subtypes, higher expression levels of FOXP3 and TGF-β are observed in MSS CRC (75), which is paralleled by the decreased number of CD8^+^ lymphocytes (76). Moreover, depletion of Tregs related to increased MSI phenotype correlated with T-cell response, which indicates the important role of Tregs to shape a suppressive TME in the MSS phenotype (77).

In recent years, the association between Tregs and prognosis of CRC patients has been controversial (78–81). This paradox may be explained by several factors. Both CD4^+^CD25^+^ natural Tregs (nTregs), which primarily suppress self-reactive T cells, and induced Tregs (iTregs), which maintain immune homeostasis, express FOXP3 during maturation (82, 83). Additionally, FOXP3 is not exclusive to Tregs, as some non-regulatory T cells and even tumor cells may also express this marker (84). Besides Foxp3, other surface markers expressed on Tregs, such as CTLA-4, LAG3, GITR, HLA-DR, ICOS, and CD127, are also essential for Treg functionality (85). These markers provide a foundation for novel strategies to improve homogeneous Treg purification. For example, based on CD45RA and HLA-DR expression, Tregs can be classified into three distinct subpopulations: naïve Tregs (CD45RA^+^, HLA-DR^−^), memory Tregs (CD45RA^−^, HLA-DR^−^), and memory/activated Tregs (CD45RA^−^, HLA-DR^+^) (86). Tregs may initially have a protective function by suppressing cancer-associated inflammation in early-stage CRC; however, they may adopt a pro-inflammatory phenotype as the cancer progresses to advanced stages (87). These findings suggest that previous studies focusing solely on FOXP3 expression may not fully capture the role of Tregs in the TME. Identifying additional biomarkers, such as HLA-DR and TGF-β, may help in isolating functionally suppressive Tregs more accurately.

#### 2.1.4 γδ T cell

In addition to the predominant CD4^+^ and CD8^+^ T cells expressing αβ TCRs, a subset of primarily CD4/CD8 double-negative T cells with γδ TCRs has garnered attention as a promising immune cell population for next-generation cancer immunotherapy. These γδ T cells can recognize antigens directly on the cell surface, independent of MHC presentation, and are found at lower expression levels in the colonic tissue of CRC patients compared to healthy controls (88–91). In CRC, two main subtypes of γδ T cells are observed: those with variable region 1 of δ chain (Vδ1), which primarily produce IFN-γ, and those expressing Vδ6, which exhibit inflammatory properties through production of IL-17A (90, 92).

In the early stages of CRC, γδ T cells display cytotoxic markers and contribute to tumor surveillance and regression through IFN-γ production. However, as the tumor progresses, tumor-infiltrating γδ T cells can acquire a pro-tumorigenic profile, characterized by IL-17 secretion (90, 93, 94). A study by Sumana et al. identified a positive correlation between the expression levels of perforin and granzymes and MSI-H status, as well as higher levels of activated memory CD4^+^ T cells, γδ T cells, and M1 macrophages, suggesting a potential antitumor role of γδ T cells in MSI-H CRC (95). Because of the diverse functional roles of γδ T cells throughout tumorigenesis, it is challenging to define their precise role in MSS and MSI CRC without taking into account tumor stage and γδ T-cell subtype. To date, differences in the function and presence of γδ T cells between MSS and MSI CRC remain largely unexplored.

#### 2.1.5 NK T cell

Apart from CD8^+^ T cell and NK cell, there is another cytotoxic subset, NK T cells, which exhibit characteristics of both conventional T cells and NK cells (96). Owing to the expression of both NK cell- and T cell-associated functional molecules, NKT cells can be activated in a T cell-like manner via recognition of glycolipids in the context of CD1d molecules and in an NK cell-like manner through inhibitory and stimulatory signals (97, 98). In addition to killing target cells directly, they are potent immune regulators by secreting TH1-, TH2-, TH17-, Treg-, and TFH cell-associated cytokines (99). Based on their TCR diversity, NKT cells can be divided into two subtypes: type I and type II NKT cells. Type I NKT cells recognize the glycosphingolipid α-galactosylceramide (α-GalCer) or its synthetic analogs, presented by MHC I-like CD1d molecules, while type II NKT cells recognize non-α-GalCer molecules presented by CD1d molecules (100). So far, it is found that type I NKT cells can be divided into five different functional subsets: TH1-, TH2-, TH17-, Treg-, and TFH-like type I NKT cells, while only two type I NKT cells were identified, TH1- and TH2-like subtypes (96). In TME, type I NKT cells generally play an antitumor role to inhibit tumor development and metastasis, while type II NKT cells associated with immunosuppression and tumor progression (101–103). CRC patients with high numbers of tumor-infiltrating type I NKT cells tend to have relatively favorable clinical outcome (104). However, tumor-infiltrating type I NKT cells exhibit impaired antitumor ability with decreased production of IFN-γ, which indicate that type I NKT cells might switch from a TH1- toward a TH2-like NKT cell subset in TME (105). Currently, owing to their variable population and function, NK T cells become a potential target to reshape TME. However, there is still an urgent need to expand and identify the NK T-cell population and the interaction between NK T cells and TME.

### 2.2 NK cells

NK cells, the cytotoxic members of the versatile family of innate lymphoid cells (ILCs), are capable of eliciting a robust and immediate antitumor response (106). Activated by cytokines such as IL-12, IL-15, and IL-18 produced by APCs, NK cells exert their antitumor effects in the TME through mechanisms including perforin/granzyme exocytosis, engagement of death receptors (such as Fas-FasL and TRAIL-TRAILR), and secretion of effector cytokines IFN-γ and TNF-α (107). In peripheral blood, nearly 90% of NK cells are characterized as CD56^dim^, CD16^+^, perforin^+^, expressing receptors such as CXCR1, CXCR2, CXCR4, and CX3CR1, which enable antibody-dependent cell-mediated cytotoxicity (ADCC) (108–110). In contrast, CD56^bright^ NK cells, which express CCR7, CXCR3, CXCR4, and CD62L, are known for producing cytokines including IFN-γ, IL-10, IL-13, TNF-β, and GM-CSF, and often exhibit protumorigenic potential (111, 112). In the TME, TGF-β—a key immunosuppressive molecule—alters the balance between NK cell subsets by downregulating chemokines associated with CD56^dim^ NK cells (CXCL1, CXCL2, CXCL1, and CXCL8) and upregulating those linked to CD56^bright^ NK cells (CXCL9, CXCL10, CCL19, and CCL5) [33,34]. Notably, neutralization of TGF-β has been shown to restore the antitumor responses of both T cells and NK cells (113, 114).

Like T cells, NK cell function is regulated by signals from activating or inhibitory receptors (115). Activating receptors include natural killer group 2-C (NKG2C), NKG2D, DNAX accessory molecule-1 (DNAM-1), CD161, and natural cytotoxicity receptors (NCRs), such as NKp30, NKp44, and NKp46. Inhibitory receptors comprise NKG2A and killer cell immunoglobulin (Ig)-like receptors, such as CD158a and CD158b (116). In CRC, NK cells exhibit reduced expression of the natural cytotoxicity receptors NKp44 and NKp46 compared to healthy controls, with functional exhaustion of NK cells correlating with shorter overall survival (116, 117). Moreover, in CRC, higher activation levels of NK cells have been positively associated with MSI status (118).

### 2.3 MDSC

MDSCs are characterized by their strong immunosuppressive role and related to poor prognosis (119). MDSCs induced by proinflammatory mediators, produced during cancer-related chronic mucosal inflammation, such as prostaglandin E2 (PGE2), IL-6, VEGF, IL-1β, S100A8/A9 proteins, and the complement component 5a (C5a) (120). Local hypoxia, low pH and exosomes secreted by tumor cells also could activate MDSCs (121–126). Moreover, IL-17 plays a crucial role in the induction, expansion, and suppressive function development of MDSCs (127). The suppressive role of MDSCs in cancer mostly contributes to the activation of two enzymes, inducible NO synthase (iNOS) and arginase-1 (ARG1), impeding proliferation and proper functioning of T cells through depletion of L-arginine (128–131). Moreover, MDSCs activate Tregs and anergy of NK cells by secretion of IL-10 and TGF-β, with TGF-β also inducing MDSCs and the epithelial-to-mesenchymal cell transition (EMT) process (132–135). MDSCs can also regulate VEGF bioavailability through inducing high levels of matrix metalloprotease 9 (MMP9) and pro-MMP9 to stimulate tumor growth and metastases in CRC (136, 137). Based on cell surface markers and cell morphology, the MDSC population can be divided into granulocytic/polymorphonuclear MDSCs (G-MDSCs/PMN-MDSCs, CD33^+^ CD11b^+^ HLA-DR^low^CD15^+^ cells) settling in the peripheral lymphoid organs, and monocytic MDSCs (M-MDSCs, CD33^+^ CD11b^+^ HLA-DR^low^CD14^+^ cells) existing in the tumor bed (138–140). PMN-MDSCs are mainly responsible for reactivating oxygen species (ROS) production, while M-MDSCs have high expression of longer activity iNOS (140, 141). In addition, enhanced infiltration of Tregs and MDSCs was detected in MSS and MSI-L tumors than its counterpart with the MSI-H phenotype (142).

### 2.4 Tumor-associated macrophages

Macrophages, a key component of the mononuclear phagocytic system (MPS), are essential for maintaining innate immune response, tissue homeostasis, and inflammation (143). Tumor-associated macrophages (TAMs) represent a major population within the immune cell component of the TME, recruited by various chemokines, including CCL2 and CCL5 and colony-stimulating factor 1 (CSF1) (144, 145). TAMs can be broadly divided into two distinct subtypes: classically activated M1 macrophages and alternatively activated M2 macrophages (146–148). M1 macrophages, which are activated by cytokines such as IFN-γ, lipopolysaccharide (LPS), and TNF-α, contribute to antitumor inflammation by promoting a TH1 response and secreting pro-inflammatory mediators, including IL-6, IL-23, and ROS (149, 150). In contrast, M2 macrophages, induced by IL-4, IL-10, IL-13 or glucocorticoids, secrete anti-inflammatory cytokines like IL-10 and IL-1β, and they play a role in promoting angiogenesis, tissue remodeling, injury repair, tumor initiation, and progression (151, 152). During the initial stages of tumor development, TAMs are primarily composed of M1 macrophages, which promote antitumor responses. However, as neoplasia progresses, there is a shift towards a predominance of M2 macrophages in the TME leading to antitumor activation, while after neoplasia formation, M2 macrophages are predominantly recruited in TME (152, 153). In CRC, tumor cells have been shown to increase the population of M2 TAMs through the PI3K/AKT signaling pathway (154, 155). Notably, M1 and M2 macrophages exhibit a degree of plasticity, with the ability to transition between states, suggesting the therapeutic potential of inducing a switch from M2 to M1 phenotypes (156, 157). Consistently, these macrophage subtypes are associated with opposite prognoses in CRC: M1 macrophage infiltration is correlated with favorable outcomes, whereas M2 TAMs are linked to poorer prognosis (158, 159). Compared to dMMR CRC, pMMR CRC shows enhanced M2 macrophage polarization, which is associated with poor survival in these cases (160). Furthermore, a recent multi-omic of MSS patients revealed that a higher density of M2 macrophage is positively correlated with improved response rate to immunotherapy (161).

### 2.5 Tumor-associated neutrophils

In human peripheral blood, neutrophils comprise 50%–70% of the circulating leukocytes and serve as the primary defense against infection (162, 163). Within TME, neutrophils are recruiting by chemokines secreted by tumor cells, notably through the CXCL1, CXCL2, CXCL5, and CXCL8 signaling axes that interact with CXCR1/2 receptors (164, 165). Tumor-derived GM-CSF can activate neutrophils and induce PD-L1 expression via the JAK/STAT3 signaling pathway (166). Neutrophils in the TME, known as tumor-associated neutrophils (TANs), can be polarized into either antitumor (N1) or protumor (N2) phenotypes, depending on the activating factors (167). N1-TANs, induced by IFN-γ, enhance tumor cytotoxicity and attenuate immunosuppression by producing TNF-α, intercellular adhesion molecule-1 (ICAM-1), ROS, and apoptosis-related factor (Fas). They also reduce the expression of arginase, a contributor to immunosuppression (167, 168). In addition, N1-TANs can release various chemokines and cytokines that stimulate immune cell proliferation and activation, thereby initiating antitumor immune responses (169). For example, TANs can recruit and activate CD8^+^ T cells by secreting CCL-3, CXCL-10, TNF-α, and IL-12 (170), and can activate NK cells and DCs through IL-18 and TNF-α release, respectively (171, 172). In contrast, N2-TANs, which are induced by TGF-β, promote tumor growth and participate in tumor migration and metastasis by upregulating arginase, MMP-9, VEGF, and additional chemokines (167, 173). As tumors progress, the N1 phenotype can convert into the N2 phenotype (174). In CRC, the prognostic value of TAN infiltration remains debated. For example, a study by Maria et al., using CD66b and myeloperoxidase (MPO) as neutrophil markers, found that higher infiltration of TANs correlated with improved prognosis and favorable response to 5-fluorouracil (5-FU)-based chemotherapy, suggesting a predominantly antitumor role for TANs (175). Conversely, other studies have identified TAN infiltration as an unfavorable prognostic marker, with higher TAN density frequently observed in MSS CRC (176–178). This paradox may be attributable to issues such as inconsistent marker selection, limited sample sizes, and unaddressed confounding factors (163).

### 2.6 Dendritic cells

DCs, as specialized professional APCs induced by Flt3L, play a pivotal role in initiating, coordinating, and amplifying antitumor immune responses (179–181). DCs encompass two primary subsets: classical/conventional DCs (cDCs) and plasmacytoid DCs (pDCs) (182, 183). The cDC subset is further divided into CD141^+^ cDC1, which presents antigens on MHC I for CD8 T cells priming by cross-presentation, and CD1c^+^ cDC2, which presents MHC II to activate CD4 T cells (184). pDCs, capable of producing large amounts of type I interferons (IFNs) during virus infection, play an essential role in maintaining immune tolerance in autoimmune conditions (185, 186). DCs enhance infiltration and toxicity of TH1, NK, and CD8^+^ T cells by enhancing their migration through CCR7 expression, upregulating co-stimulatory molecules such as CD80, CD83, and CD86, and secreting of pro-inflammatory cytokines like IL-12, IL-6, TNF-α, and IL-1β (187–190). However, within TME, DC function is frequently impaired by immunosuppressive signals from tumor cells. This impairment is a key contributor to immune evasion, tumor growth, metastasis initiation, and treatment resistance in various cancers, including CRC (187, 188, 191–195). In TME, TGF-β, TNF-α, IDO-1, PGE2, IL-6, IL-10, VEGF, and GM-CSF hinder DC migration, antigen presentation, and the effective activation of T cell and NK (196, 197). In CRC specifically, the density of mature DCs ranks lowest in metastatic sites, intermediate in primary tumor sites, and highest in normal mucosa (198, 199). In addition, mature DCs are typically localized in the invasive margin and form clusters with T cells with tertiary lymphoid structures (TLS), whereas immature DCs are more dispersed through the tumor stroma, underscoring the suppressive effects of TME on DC maturation and function (200, 201). Recent findings have also highlighted a deficiency of activated T cells and DCs in MSS CRCs and liver metastasis, where enhanced DC infiltration combined with immune checkpoint blockade (ICB) treatment significantly improved survival in murine models (202).

## 3 Current therapeutic strategies

Since initial immunotherapy trials in CRC revealed substantial efficacy differences between MSS and MSI-H subtypes, research efforts have largely pivoted to focus on the MSS subtype. A landmark phase 3 open-label trial, KEYNOTE-177, compared the efficacy of pembrolizumab to standard chemotherapy in patients with MSI-H and dMMR CRC, demonstrating a significantly improved progression-free survival (PFS) in the immunotherapy group (16.5 vs. 8.2 months). This trial marked a milestone in CRC immunotherapy, establishing pembrolizumab as the new first-line treatment for patients with MSI-H/dMMR mCRC (203). Additionally, the combination of CTLA-4 and PD-1 inhibitors has been shown to improve survival in patients with MSI-H/dMMR mCRC and has been approved as a second-line treatment option (204). In contrast, immunotherapy attempts in MSS CRC have yielded unsatisfactory outcomes. As the MSS phenotype represents the majority of CRC cases, numerous strategies are being explored to enhance immunotherapy efficacy in MSS CRC. The current clinical trials investigating these approaches are summarized in **Table 2**.

**Table 2:** Current clinical trials for MSS CRCs.

Study	Treatment	Phase	Patient	Primary endpoint	Status
NCT04262687	Capecitabine + Oxaliplatin + Bevacizumab + Pembrolizumab	II	MSS mCRC with high immune infiltrate	PFS	Recruiting
NCT05914389	Capox ± Envafolimab ± *Clostridium butyricum* + Colectomy	II	Locally advanced MSS CRC	TRG0/1	Recruiting
NCT05359393	A combination therapy including tislelizumab	II	MSS rectal cancer With resectable liver/pulmonary metastasis	NED rate	Not yet recruiting
NCT05576480	SCRT + Penpulimab + CAPEOX	II	MSS locally advanced rectal cancer	pCR	Not yet recruiting
NCT05160727	Radiotherapy + Tislelizumab + Irinotecan	II	MSS/PMMR mCRC	ORR	Recruiting
NCT04659382	XELOX + Bevacizumab + Atezolizumab + SIRT	II	MSS/PMMR mCRC	PFS	Recruiting
NCT04030260	Regorafenib + PD-1 antibody + Radiotherapy	II	MSS/PMMR mCRC	PFS	Recruiting
NCT03104439	Nivolumab + Ipilimumab + Radiation	II	MSS/MSI CRC	DCR	Recruiting
NCT03608046	Avelumab + Cetuximab + Irinotecan	II	MSS mCRC	ORR	Recruiting
NCT05409417	Tislelizumab + XELOX and Bevacizumab/FOLFOX and Cetuximab	II/III	MSS CRC with liver metastasis	Conversion rates, R0 resection rate, safety	Recruiting
NCT04745130	Sintilimab and regofinib ± cetuximab	II	MSS/MSI-L/PMMR CRC	OS	Recruiting
NCT04362839	Regorafenib + Ipilimumab + Nivolumab	I	MSS mCRC	DLT	Active
NCT05382741	Adjuvant Durvalumab+ Regorafenib/observation	II	Stage IV CRC	DFS	Recruiting
NCT04963283	Cabozantinib + Nivolumab	II	MSS mCRC	DCR	Recruiting
NCT03170960	Cabozantinib + Atezolizumab	I/II	Metastatic solid tumor	MTD, ORR	Active
NCT03539822	Cabozantinib + Durvalumab ± Tremelimumab	I/II	Gastrointestinal malignancies	MTD, ORR	Recruiting
NCT03668431	PDR001 + Dabrafenib + Trametinib	II	mCRC with BRAF V600E mutation	ORR, safety	Recruiting
NCT03374254	Pembrolizumab + mFOLFOX7 ± Binimetinib	I	mCRC	DLT	Active
NCT04294160	Dabrafenib + LTT462 ± Trametinib/LXH254/TNO155/Spartalizumab/Tislelizumab or Dabrafenib + trametinib + TNO155	I	mCRC with BRAF V600E mutation	DLT, safety	Active
NCT03711058	Copanlisib + Nivolumab	I/II	Relapsed/refractory MSS CRC	DLT/ORR	Active
NCT05627635	FOLFOX + Bevacizumab + Botensilimab + Balstilimab	I/II	MSS mCRC	ORR, safety	Recruiting
NCT03442569	Nivolumab + Ipilimumab + Panitumumab	II	MSS mCRC	ORR	Active
NCT03642067	Nivolumab + Relatlimab	II	Advanced MSS CRC	ORR	Recruiting
NCT05731726	Serplulimab + CAPEOX + Celecoxib	II	Advanced MSS/MSI-L/PMMR CRC	pCR rate	Recruiting
NCT03800602	Nivolumab + Metformin	II	Refractory MSS CRC	ORR	Active
NCT05243862	PolyPEPI1018 + Atezolizumab	II	MSS mCRC	Safety	Recruiting
NCT05141721	Fluoropyrimidine + Bevacizumab ± Atezolizumab Ipilimumab GRT-C901/GRT-R902 (Neoantigen Vaccine)	II/III	MSS mCRC	ctDNA decrease, PFS	Recruiting
NCT05733611	RP2/RP3 + Atezolizumab + Bevacizumab	II	Advanced MSS/PMMR CRC	ORR	Not yet recruiting
NCT04301011	TBio-6517 ± Pembrolizumab	I/II	MSS CRC	ORR, safety	Active
NCT05061017	Nivolumab + Pixatimod	II	MSS mCRC	ORR	Recruiting
NCT05279677	FMT + Sintilimab + Fruquintinib	II	Advanced CRC	ORR	Recruiting
NCT04729322	FMT + Pembrolizumab/Nivolumab	II	PD-1 non-responding dMMR CRC	ORR	Recruiting

### 3.1 Combination with chemotherapy

Chemotherapy, a cornerstone of cancer treatment, has been shown to impact immune cells directly, in addition to its cytotoxic effects on malignant cells. In the murine model, cyclophosphamide appears to enhance TH1 differentiation and reduce Treg by mediating an IRF1-dependent, IL-12-mediated response (205). Agents like 5-fluorouracil (5-FU) and gemcitabine have been found to selectively decrease the frequency of MDSCs in the spleen and tumor bed, without directly affecting T cells, DCs, NK cells, or B cells. The elimination of MDSCs, in turn, promotes IFN-γ production by infiltrating CD8^+^ T cells (206). Furthermore, cyclophosphamide, paclitaxel, and doxorubicin can repolarization of macrophages from an M2 to an M1 phenotype, shifting them towards a more pro-inflammatory, antitumor function (207, 208). Chemotherapy also exerts differing effects on TME of primary and metastatic lesions. An analysis of immune profiles in primary tumor and paired metastasis tissue samples from patients with MSS CRCs revealed that increased expression of CD8 and PD-L1 was higher in metastatic sites following neoadjuvant FOLFOX treatment (folinic acid, 5-FU, and oxaliplatin) (209). Beyond MSI CRCs, studies indicate that a subset of MSS CRCs exhibit high CD8^+^ infiltration, suggesting potential sensitivity to immunotherapy (38). A phase II clinical trial (NCT04262687) is currently investigating the efficacy of PD-1 inhibitors in patients with high immune infiltration. However, for the majority of MSS CRC cases, the combination of PD-1 inhibitors and chemotherapy has shown limited effectiveness. Recent research trends are exploring the potential of this combination in neoadjuvant settings, as seen in clinical trials NCT05914389, NCT05359393, and NCT05576480.

### 3.2 Combination with radiotherapy

Radiotherapy also plays a pivotal role in remodeling antitumor immunity and enhancing immunotherapy efficacy. Studies have shown that radiation exposure can induce M1 polarization, enhance NK cell infiltration, and reduce TGF-β levels, collectively contributing to an immune-stimulatory environment (210–212). In the VOLTAGE-A phase I b/II study (NCT02948348), neoadjuvant chemoradiotherapy (CRT) followed by nivolumab and radical surgery was tested in patients with MSS locally advanced rectal cancer, with 30% of patients achieving a pathologic complete response (pCR), which show a great potential of this combination (213). Post-surgical pathological analysis revealed that a PD-L1 tumor proportion score (TPS) of ≥1% and a CD8^+^ T cell-to-effector Treg ratio of ≥2.5 were associated with a higher pCR rate. Current clinical trials are further investigating the efficacy of combining radiotherapy with PD-1 monoclonal antibody therapy in patients with metastatic CRC (NCT05160727, NCT04659382, NCT04030260, and NCT03104439).

### 3.3 Combination with anti-angiogenic agent

VEGF, known for promoting tumor vessel growth, also plays an immunosuppressive role by increasing the recruitment of Tregs and MDSCs and inhibiting the differentiation and activation of DCs. Antiangiogenic agents targeting the VEGF/VEGFR pathway, such as bevacizumab, can help mitigate VEGF-associated immunosuppression (214). In the phase II AtezoTRIBE trial, patients with metastatic CRC received FOLFOXIRI plus bevacizumab with or without the addition of atezolizumab. In the pMMR subgroup, disease progression was observed in 81% of patients in the control group versus 70% in the atezolizumab group, indicating a potential synergistic effect between atezolizumab and bevacizumab (215). However, another phase II trial comparing mFOLFOX6 plus bevacizumab alone or with avelumab, showed no significant difference in median PFS between experimental and control groups (216).

### 3.4 Combination with an anti-EGFR agent

Cetuximab, a chimeric IgG1 monoclonal antibody targeting EGFR, is a first-line treatment for advanced CRC. Recent studies have shown that cetuximab administration can increase CD3^+^ T, CD8^+^ T, and NK cells, while decreasing Tregs (217). The phase II AVETRIC trial investigated the efficacy and safety of first-line mFOLFOXIRI combined with cetuximab and avelumab in RAS wild-type mCRC patients, reporting an impressive 98% disease control rate (DCR) at the ASCO meeting, highlighting the potential of cetuximab combined with immunotherapy. A phase II study (NCT03608046) is currently evaluating avelumab combined with cetuximab and irinotecan for patients with refractory MSS CRC. Additionally, clinical trials (NCT05409417 and NCT04745130) are comparing the efficacy of cetuximab with anti-angiogenic agents (bevacizumab and regorafenib) in combination with immunotherapy for CRC patients.

### 3.5 Combination with tyrosine kinase inhibitors

Regorafenib is a multi-targeted small-molecule tyrosine kinase inhibitor approved for the treatment of refractory and mCRC. It achieves antitumor effects by inhibiting various tyrosine kinases involved in tumor growth, angiogenesis, and metastasis. In the phase Ib trial REGONIVO, 25 patients with CRC (24 pMMR-MSS and 1 dMMR-MSI-H) received a combination of nivolumab and regorafenib, achieving an objective response rate (ORR) of 36% and a PFS of 7.9 months (218). However, in a subsequent phase II clinical trial of the same combination in 70 patients with pMMR/MSS CRC, ORR dropped to 7%, with a PFS of just 1.8 months (219). Another phase Ib/II study combining regorafenib and toripalimab reported an improved ORR of 15.2% and a DCR of 36.4% (220). Given the limited efficacy of these combinations, researchers are now exploring regorafenib in conjunction with two ICIs—nivolumab and ipilimumab (NCT04362839)—and as part of adjuvant therapy (NCT05382741) to assess potential improvements in treatment outcomes.

Cabozantinib, a small-molecule multi-tyrosine kinase inhibitor targeting kinases such as VEGFR2, MET/HGF, AXL, MER, and TYRO3, has demonstrated antitumor activity in the preclinical CRC model. It enhances the immune environment by reducing the function of Tregs and increasing cytokine production (221–223). In the CAMILL trial, 17 patients with MSS CRC were treated with a combination of cabozantinib and durvalumab, showing potential efficacy with ORR of 23.5% and a DCR of 88.2%. The median PFS was 4.6 months, and the OS reached 9.6 months (224).

The RAS/BRAF/MEK/ERK pathway, which operates downstream of various growth factor receptors, including EGFR, is often overexpressed and activated in CRC, particularly in cases with dysregulated MAPK pathway signaling (225). Studies have shown that dysregulation in this pathway is linked to reduced T-cell infiltration, activation of MDSCs, DC function, and lower expression of inhibitory molecules such as CTLA4, PD-L1, PD-L2, LAG3, and TIM3, contributing to an immunosuppressive TME (226, 227). These findings suggest a potential synergy between immunotherapy and selective RAS/BRAF/MEK/ERK pathway inhibitors in patients with pMMR/MSS mCRC. However, a phase III trial (COTEZO) evaluating the combination of atezolizumab and MEK inhibitor cobimetinib versus atezolizumab alone or regorafenib in patients with chemo-refractory CRC did not meet its primary endpoint, showing no significant difference in PFS or ORR between the treatment arms (228). Ongoing clinical trials are now assessing the efficacy of PD-1/PD-L1 inhibitors combined with MEK inhibitors (NCT03668431, NCT03374254, and NCT04294160).

### 3.6 Combination with a PI3K inhibitor

The PI3K/Akt pathway is frequently activated in various cancers and contributes to carcinogenesis by promoting cell proliferation, survival, metabolic reprogramming, invasion, and metastasis, while inhibiting autophagy and senescence. Recent studies have indicated that PI3K/Akt pathway status can significantly influence the immune microenvironment (229). In patients with CRC, mutations in the PI3K/Akt pathway have emerged as an independent predictor of immunotherapy response, correlating to better OS and increased immune cell infiltration, such as M1 macrophages, neutrophils, and NK cells, within TME (230). Copanlisib, a pan-class I PI3K IV inhibitor, has shown promising preclinical antitumor activity in CRC (231). A phase I/II clinical trial (NCT03711058) is currently underway to evaluate the efficacy and safety of combining copanlisib with nivolumab in patients with relapsed/refractory MSS CRC.

### 3.7 Combination with novel ICIs

CTLA-4, another checkpoint, is expressed on the surface of activated T cells, where it competes with CD28 receptors for binding to B7 ligands on APCs, leading to anergy in T cells (232). CTLA-4 expression is primarily regulated by FOXP3 on Tregs, thereby contributing to an immunosuppressive TME (233). The combined blockade of PD-1 and CTLA-4 has shown synergistic clinical benefit, resulting in regulatory approval for conditions such as metastatic melanoma, advanced renal cell carcinoma, and metastatic CRC with the MMR/MSI-H phenotype (234). The CCTG CO.26 study was the first to demonstrate that anti-PD-L1 combined with anti-CTLA-4 could extend OS in patients with refractory MSS CRC compared to best supportive care (BSC) (235). Ongoing clinical trials are assessing the efficacy of combining PD-1 and CTLA-4 blockade with additional treatments, such as FOLFOX and bevacizumab (NCT05627635) or panitumumab (NCT03442569).

LAG-3 is an inhibitory signaling expressed on both CD4^+^ and CD8^+^ T cells upon antigen simulation (236). It is constitutively expressed on FOXP3^+^ Tregs, resulting in elevated levels of immunoregulatory cytokines IL-10 and TGF-β, which suppress tumor-specific T-cell responses (236). Preclinical studies indicate that dual blockade of LAG-3 and PD-1 can restore T-cell function and reverse anergy. In a phase I clinical trial, the combination of an anti-LAG-3 antibody (favezelimab) and an anti-PD-1 antibody (pembrolizumab) in patients with advanced MSS mCRC demonstrated promising antitumor ability with an OS of 8.3 months and a median PFS of 2.1 months (237). Currently, a phase II study (NCT03642067) is underway to assess the safety and efficacy of nivolumab in combination with relatlimab in patients with advanced MSS CRC.

### 3.8 Combination with other drugs

Temozolomide (TMZ) is an oral alkylating agent methylating the O^6^ position of guanine in DNA, providing therapeutic efficacy in several solid tumors such as glioma, glioblastoma, neuroendocrine tumors, melanoma, and sarcomas. O^6^-methylguanine methyltransferase (MGMT), a DNA repair enzyme encoded by the MGMT gene, plays a crucial role in repairing DNA damage caused by alkylating agents and is associated with the reduced therapeutic efficacy of TMZ (238). Epigenetic silencing of MGMT, primarily through methylation of its promoter region, is observed in approximately 40% of CRC cases (239). The ORR of patients with MGMT-methylated mCRC treated with TMZ was 12% (240). Acquired resistance to TMZ is often linked to hypermutation and the emergence of mutations in MMR genes, particularly MSH6 (239). The ARETHUSA trial (NCT03519412) analyzed tissue and circulating tumor DNA (ctDNA) in patients with MGMT-deficient, MMR-proficient, RAS-mutant mCRC treated with TMZ priming. Findings showed increased TMB and MSH6 mutation in 94% of cases post-TMZ treatment. A subset of patients with TMB > 20 mutations per megabase, subsequently treated with pembrolizumab, achieved disease control (238). These promising results suggest that TMZ could serve as a priming agent for re-sensitizing pMMR/MSS CRCs to ICIs. Supporting these findings, the phase II MAYA trial (NCT03832621) administrated two cycles of TMZ followed by a combination of ipilimumab and nivolumab in patients with chemo-refractory MSS CRC and MGMT silencing, yielding an improved ORR of 45%, an 8-month PFS rate of 36%, and an OS of 18.4 months (239).

Cyclooxygenase enzyme-2 (COX-2) expression is significantly elevated in 79% of MSS CRC, compared to only 48% of MSI CRC and is associated with poorer OS (241, 242). Prostaglandin E2 (PGE2), a product of COX-2, can activate MDSCs via STAT3 phosphorylation, leading to suppressed CD8^+^ T-cell proliferation, reduced cytotoxicity against tumor cells, and impaired macrophage phagocytosis of cancer cells through the EP4-PI3K-Akt-ND-1 signaling pathway (243–250). Preclinical studies suggest that COX inhibitors could serve as effective adjuvant for ICIs, potentially reactivating immunosuppressive TME in MSS CRCs (251). The NICHE study (NCT03026140) explored the neoadjuvant administration of ipilimumab and nivolumab, with or without celecoxib, in patients with pMMR CRC. Results showed that 4 of 15 patients (27%) in the non-celecoxib group experienced pathological responses, whereas 4 of 7 patients (57%) in the celecoxib group achieved pathological responses (252). An ongoing phase II study (NCT05731726) is investigating the role of celecoxib as part of a neoadjuvant treatment regimen combined with PD-1 monoclonal antibody and chemotherapy in locally advanced CRC patients with the pMMR/MSS phenotype.

Accumulating evidence shows that metformin, a medication primarily used to treat type II diabetes, may regulate tumor cell metabolism through reducing their oxygen consumption, alleviating tumor hypoxia, and supporting the activity of cytotoxic T lymphocytes within tumor tissues (253). A phase II clinical trial (NCT03800602) is investigating the efficacy of combining metformin with nivolumab in the treatment of refractory MSS CRC.

### 3.9 Combination with a bispecific antibody

A bispecific antibody is engineered to recognize two distinct antigens, effectively bridging the tumor cell and the T cell to bolster immune activity against tumor cells (254). Cibisatamab (RO6958688), the most extensively studied bispecific antibody in CRC, targets CEA on colorectal tumor cells and CD3 on T cells, promoting increased T-cell infiltration, activation, and PD-1/PD-L1 upregulation (255). Preclinical studies have demonstrated that combining PD-1/PD-L1 blockade with an anti-CEA/CD3 bispecific antibody can significantly enhance T cell-mediated antitumor activity (256). A phase I study comparing the efficacy of anti-CEA/CD3 bispecific antibody as monotherapy versus in combination with atezolizumab (NCT02650713) in patients with mCRC showed that combination therapy notably improved outcomes, particularly in patients with MSS CRC (257).

### 3.10 Combination with vaccines

Cancer vaccines aim to stimulate an antitumor response by isolating and presenting tumor-specific antigens. DCs, as pivotal APCs, are considered promising vectors for cancer vaccines. However, two phase II clinical trials assessing the efficacy of an autologous tumor lysate DC vaccine—both as monotherapy and in combination with avelumab—in patients with MSS CRC demonstrated that while the vaccine induced a tumor-specific immune response, it did not yield significant clinical benefits (258, 259).

Granulocyte-macrophage colony-stimulating factor (GM-CSF) is an important growth and differentiation factor for DCs. The GVAX colon vaccine, composed of GM-CSF-expressing irradiated tumor cells, is designed to induce T-cell immunity (260). Preclinical studies have demonstrated that dual blockade of PD-1 and CTLA-4, combined with the GM-CSF gene-transfected GVAX vaccine, effectively induced tumor rejection and restored T-cell activity in CRC murine models (261). In a phase II study (NCT02981524), the efficacy of GVAX combined with pembrolizumab was assessed in patients with pMMR CRC; although it failed to meet its primary objective, with no objective responses observed, biochemical responses (≥30% decline in CEA) were achieved in 7 out of 17 patients (41%) (262).

PolyPEPI1018 is a cancer vaccine consisting of six synthetic peptides derived from seven tumor-associated antigens (TAAs) commonly expressed in CRCs. In OBERTO-101 study, PolyPEPI1018 was administered as an interim therapy between first-line induction and maintenance treatment in 11 previously treated patients with MSS mCRC, resulting in an increased density of tumor-infiltrating lymphocytes (TILs) and a broad antitumor T-cell response (263). Following these promising results, a phase II clinical study (NCT05243862) is currently recruiting to further investigate the safety and efficacy of combining PolyPEPI1018 with atezolizumab.

Neoantigens are tumor-specific antigens produced by somatic mutations, and personalized neoantigen vaccines have demonstrated the ability to induce robust antitumor immune responses across multiple cancer types, including MSS CRC (264). When combined with PD-1 blockade, these vaccines produce a synergistic effect by enhancing neoantigen-specific CD4^+^ and CD8^+^ T-cell responses (265, 266). The efficacy of combining personalized neoantigen vaccines with immunotherapy for MSS CRC is currently under investigation in a clinical trial (NCT05141721).

### 3.11 Combination with an oncolytic virus

Oncolytic viruses (OVs), such as adenovirus, vaccinia virus, and herpes simplex virus, are genetically modified to selectively or preferentially infect cancer cells, resulting in direct lysis of tumor cells and induction of an antitumor immune response (267). Moreover, OVs engineered with cytokines like GM-CSF, IL-15, and TNF superfamily members can enhance this immune response by activating immune cells, including CD4^+^ and CD8^+^ T cells, and increasing the expression of perforin and granzyme B in the TME. This process effectively transforms “cold” tumors into “hot” ones (268–270). The combination of OVs with ICIs has shown synergistic effects in preclinical CRC models (271, 272). A phase 1b clinical study of OV (Pexa-Vec) combined with ICIs in patients with CRC demonstrated an acceptable safety profile and achieved a 67% radiographically stable disease rate, resulting in further trails to evaluating the efficacy of Pexa-Vec (273). Two additional clinical studies are ongoing to further evaluate the efficacy of this combination (NCT05733611 and NCT04301011).

### 3.12 Combination with other therapies

Toll-like receptor 9 (TLR9), a pattern recognition receptor, is expressed on B cells and plasmacytoid DCs in humans (274). Pixatimod, a TLR9 pathway activator, inhibits the infiltration of TAMs, stimulates DCs, and activates NK cells (275). A phase Ib clinical trial evaluated the safety and efficacy of pixatimod combined with nivolumab in 25 participants with microsatellite-stable (MSS) metastatic CRC (mCRC), achieving PR in 3 participants and SD in 8 participants, which meets its primary endpoint and warrants further investigation (276). A phase II clinical trial (NCT05061017) is currently recruiting to further investigate this combination in MSS mCRC.

Fecal microbiota transplantation (FMT) offers a promising approach to modify the gut microbiome with a favorable safety profile. Preclinical studies have shown that microbiome manipulation can enhance the antitumor activity of ICIs, likely due to alterations in glycerophospholipid metabolism and increased expression of IFN-γ and IL-2 in TME (277). Two clinical trials (NCT05279677 and NCT04729322) are currently investigating the efficacy of combining FMT with ICIs.

## 4 Conclusion

Despite considerable advancements in immunotherapy, specifically ICIs, the efficacy of these treatments remains limited for the majority of CRC patients, especially those with MSS and pMMR tumors. Given that MSS/pMMR CRC represents a substantial proportion of cases, there is an urgent need to develop novel therapeutic strategies. The TME plays a significant role in determining treatment responses, with variations in immune cell composition and activity affecting the efficacy of ICIs in MSS CRC.

As we have discussed, CRCs exhibit distinct TME profiles depending on their MSI status. MSI CRCs tend to have an inflammatory TME, characterized by a significant increase in plasma cells, CD8^+^ T cells, activated memory CD4+ T cells, follicular T helper cells, NK cells, M1 macrophages, and neutrophils, alongside a marked reduction in Tregs (278). Additionally, the TME of MSI CRCs shows higher levels of inflammatory cytokines, including TNF-α, perforin, granzyme, IL-1, IL-6, and IFN-γ (279). Some components of the TME, such as TH17 cells, Tregs, γδ T cells, TAMs, TANs, and NK T cells, exhibit dual functions depending on the specific context, contributing to the complex network of interactions within the TME (92, 152, 153, 167, 280). Furthermore, the TME is highly dynamic throughout CRC progression and metastasis. For example, TAMs initially exhibit an inflammatory profile dominated by antitumor M1 macrophages, but over time, as the tumor progresses, the TME shifts to a more pro-tumorigenic environment with an increased presence of M2 macrophages (152, 153). The roles and properties of individual TME components can change significantly during tumor progression and in response to treatment. Given the dynamic nature of the TME, comprehensive and functional characterization of its components at different stages of tumor development is crucial. This will help identify CRC patient subgroups most likely to benefit from TME-modulating therapies and determine the optimal timing for these treatments.

Recent studies underscore the potential of combination therapies involving ICIs and traditional treatments such as chemotherapy and radiotherapy, as well as targeted approaches such as tyrosine kinase inhibitors, anti-angiogenic agents, and EGFR inhibitors, to re-sensitize MSS CRC to immunotherapy. Emerging strategies incorporating bispecific antibodies, cancer vaccines, OVs, and fecal microbiota transplantation (FMT) also show promise in enhancing immune responses within the TME, converting it from an immunosuppressive to an immunostimulatory state. Personalized approaches, including neoantigen vaccines and other novel ICIs targeting checkpoints like CTLA-4 and LAG-3, may further optimize treatment efficacy by addressing specific immune evasion mechanisms.

While these approaches hold promise, clinical efficacy and long-term outcomes for MSS/pMMR CRC patients remain to be conclusively determined. Future research and clinical trials will be essential to better understand the complex interactions within the TME and to develop integrated, personalized therapeutic strategies that leverage the latest advancements in immuno-oncology.
